# Synthesis and Properties of Trehalose-Based Flexible Polymers Prepared from Difurfurylidene Trehalose and Maleimide- Terminated Oligo(dimethylsiloxane) by Diels-Alder Reactions

**DOI:** 10.3390/ma3010369

**Published:** 2010-01-12

**Authors:** Naozumi Teramoto, Masashi Niwa, Mitsuhiro Shibata

**Affiliations:** Department of Life and Environmental Sciences, Faculty of Engineering, Chiba Institute of Technology, 2-17-1 Tsudanuma, Narashino, Chiba 275-0016, Japan

**Keywords:** trehalose, poly(dimethylsiloxane), Diels-Alder reaction, furfural, renewable resources

## Abstract

Difurfurylidene trehalose (DFTreh) was synthesized from trehalose and furfural by an acetalization reaction. Maleimide-terminated dimethylsiloxane oligomers (DMS-BMI) were synthesized from amine-terminated dimethylsiloxane oligomers by condensation with maleic anhydride. Three types of DMS-BMI with different length were prepared. Trehalose-based polymers were synthesized by Diels-Alder reaction of DFTreh and DMS-BMI. The reaction proceeded at 40~70 °C to produce a polymer with a maximum weight average molecular weight of ~19,000. The thermal degradation temperature increased with the increase of the length of the oligo(dimethylsiloxane) units. The differential scanning calorimetry (DSC) measurements revealed the glass transition temperature (*T*_g_) of the polymer at -130~-120 °C, and no distinct *T*_g_ not observed above room temperature in the DSC measurement. The polymer products are not liquid at room temperature, and solid films can be obtained by casting from solution, implying a phase-separated structure made up of soft and hard segments. The phase-separated structure was confirmed by transmission electron microscope (TEM) study. The DSC curve of the polymer showed a broad endothermic peak at 110~160 °C, suggesting that a retro-Diels-Alder reaction occurred. When a *N,N*-dimethylformamide solution of the polymer was kept at 100 °C and the resulting solution was analyzed by gel permeation chromatography (GPC), the molecular weight decreased and monomers appeared.

## 1. Introduction

Polymers from renewable resources and resources found in abundance are favorable as substitutes for common polymers derived from petroleum. Carbohydrate polymers are very attractive as polymeric materials derived from renewable resources, but most of them have rigid main chains because of rigidity of cyclic structures and intermolecular interactions such as dipolar-dipolar interaction [1,2,3]. Even if we reduce the intermolecular interactions by chemical modification, their main chains remain rigid and the glass transition temperatures would not be reduced much. To overcome this fundamental problem, we must modify the main chain and control its rigidity. In the previous studies, we have proposed disaccharide-based linear polymers and demonstrated their syntheses [4,5,6,7,8]. Firstly, we prepared a polyacetal from trehalose and terephthalaldehyde [4]. However, the glass transition temperature of this polymer was very high (224 °C), because its chain is very rigid. Then we synthesized a trehalose-based polymer containing alkyl groups from a diepoxytrehalose derivative and *N,N'*-dimethyldiaminohexane, and found that the glass transition temperature of the polymer is moderate (~100 °C) [5]. However, it is hard to process this polymer into films, because of its brittleness. Most recently, siloxane oligomer units were introduced into a trehalose-based polymer chain by hydrosilylation reactions to obtain flexible polymers which can be processed into films [7].

In our strategy for the synthesis of disaccharide-based linear polymers, we have consistently used trehalose as disaccharide. Trehalose is very interesting saccharide found widely distributed in organisms and it plays important roles in biopreservation [8,9,10,11]. Since Hayashibara Co. Ltd. established an inexpensive productive method using enzymes [12], production costs have been reduced greatly and the application fields have been consequently extended. In this method, starch is the starting material and most reactions proceed under mild conditions and are therefore environmentally benign.

The Diels-Alder reaction is a well-known cycloaddition reaction between dienophiles and dienes. The reaction is usually not influenced by the presence of many functional groups such as amine, hydroxyl, carboxyl and so on. In the previous study, the Diels-Alder reaction was applied in polymerization of difurfurylidene trehalose [6]. Since 4,4'-bismaleimidodiphenylmethane and the 1,6-bismaleimidohexane were used as dienophiles, the polymer chains are not flexible enough to form a film. Nonetheless liquefaction of the polymer started at 140 °C. The reaction of a furan derivative as a dienophile compound with a maleimide derivative as a diene compound is typical of the Diels-Alder reaction, and the addition product is known to be degraded into starting components by heating, *i.e.,* by the retro-Diels-Alder reaction. Therefore, the Diels-Alder reaction is often used for chain extension and crosslinking of polymers [13,14,15,16,17,18,19,20,21,22,23,24] for the purpose of easy processing and recyclability.

**Scheme 1:**
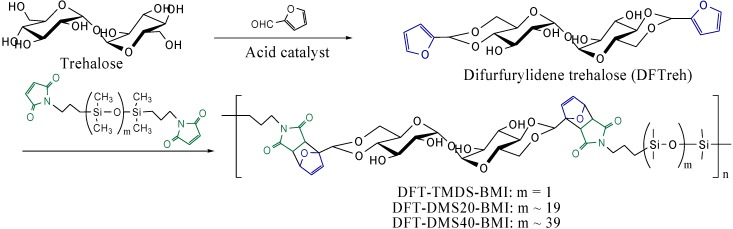
Synthesis of polymers from difurfurylidene trehalose and bismaleimide-terminated dimethylsiloxane oligomers by Diels-Alder reaction.

As described above, we reported the synthesis of trehalose-based rigid polymers by the Diels-Alder reaction in the previous study [6]. Under the inspiration of flexible polymers [7], we designed a trehalose-based polymer containing siloxane oligomer units synthesized by the Diels-Alder reaction in the present study (Scheme 1). Poly(dimethylsiloxane) is a very flexible amorphous polymer whose glass transition temperature is lower than -100 °C, and usually it is a viscoelastic liquid at room temperature without crosslinking. In the previous study, we found that the trehalose-based linear polymers containing oligo(dimethylsiloxane) units synthesized by the hydrosilylation reaction are in a solid state at room temperature [7]. We considered that the dibenzylidene trehalose units acted as a rigid segment with ability of intermolecular hydrogen bond formation. In the present study we synthesized solid polymers with flexible chains between rigid trehalose units by the Diels-Alder reaction.

## 2. Experimental

### 2.1. Materials

α,α-d-Trehalose dihydrate was kindly provided by Hayashibara Co., Ltd. (Japan) and was dehydrated at 130 °C for 24 h before use. Furfural, 1,1,1,3,3,3-hexamethyldisilazane (HMDS), and 1,3-bis(3-aminopropyl)tetramethyldisiloxane (TMDS-BA) were used as received from Tokyo Chemical Industry Co., Ltd. (Tokyo, Japan). *N,N*-dimethylformamide (DMF; anhydrous) and a telechelic amine-terminated dimethylsiloxane oligomer with the molecular weight of *ca.* 3,000 (DMS40-BA) were used as received from Aldrich Chemical Company (WI). A telechelic amine-terminated dimethylsiloxane oligomer with the molecular weight of *ca.* 1,500 (DMS20-BA) was used as received from Gelest, Inc. (PA, USA). The amount of the amine groups in dimethylsiloxane oligomers was determined by the integral values of proton signals in the ^1^H-NMR spectrum. Other reagents were purchased from Kanto Kagaku (Tokyo, Japan).

### 2.2. Synthesis of 4,6,4’,6’-O-difurfurylidene-α,α-d-trehalose (DFTreh)

Synthesis of DFTreh was carried out as reported previously [6]. Dried trehalose (6.84 g, 20 mmol) was dissolved in 60 mL of hot anhydrous DMF in a three-neck round-bottom flask equipped with a Dean-Stark trap. To the solution were added 40 mL of furfural (480 mmol), 7 g of Amberlyst 15 (33 mg-equivalent), and 20 mL of anhydrous toluene. The solution was constantly stirred and toluene was refluxed at 70 °C for 24 h under reduced pressure (100 torr). After the reaction, the solvent was evaporated and 100 mL of 5% aqueous sodium hydrogen carbonate solution was added. The insoluble part obtained was dried at room temperature in *vacuo* for 24 h, dissolved in 5 mL of DMF and precipitated in 200 mL of ethyl acetate. The precipitate was dried at room temperature in *vacuo* for 24 h to yield a brown solid (4.11 g, 41% yield). IR (KBr): 3,400 (OH), 3,150 (aromatic CH), 2,950, 2,900 (aliphatic CH), 1,500 (aromatic C=C), 1,080, 990 (acetal, C-O), 750 cm^-1^ (aromatic CH); ^1^H- NMR (DMSO-*d_6_*, 400 MHz) *δ* = 7.64 (q, 2H, furan), 6.49 (d, 2H, furan), 6.46 (d, 2H, furan), 5.65 (s, 2H, acetal-*H*), 5.31 (d, 2H, O*H-2*), 5.24 (d, 2H, O*H-3*), 4.92 (d, 2H, *H-1*), 4.05 (m, 4H, *H-5, H-6-1*), 3.75 (m, 2H, *H-3*), 3.64 (m, 2H, *H-6-2*), 3.40 (m, overlapping with resonance of water, 4H, *H-2, H-4*).

### 2.3. Synthesis of maleimide-terminated dimethylsiloxane oligomers (DMS-BMI)

Maleimide-terminated dimethylsiloxane oligomers were synthesized by the method for preparation of bismaleimide according to the previous article reported by Kamahori *et al.* [25]. Maleic anhydride (3.46 g, 35 mmol) was dissolved in 96 mL of benzene, and to this solution was added the amine-terminated dimethylsiloxane oligomer (16 mmol) in 64 mL of benzene. Three types of the amine-terminated dimethylsiloxane oligomer with different length (TMDS-BA, DMS20-BA and DMS40-DA) were used. After the mixture was stirred at room temperature for 1 h, ZnBr_2_ (7.94 g, 35 mmol), HMDS (10 mL, 80 mmol), and benzene (19 mL) was added. The reaction was carried out by stirring under reflux for 2 h. After the reaction, the solution was diluted with 192 mL of ethyl acetate and washed with 128 mL of 0.5 N HCl, 96 mL of saturated NaHCO_3_ in twice, and 96 mL of saturated NaCl in twice. The organic layer was dried over Na_2_SO_4_ for 24 h; Na_2_SO_4_ was filtered off; and solvent was removed by a rotary evaporator to yield a pale brown liquid. The products from TMDS-BA, DMS20-BA and DMS40-BA were denoted as TMDS-BMI (5.8 g, 89% yield), DMS20-BMI (15.7 g, 61% yield) and DMS40-BMI (26.0 g, 52% yield), respectively. For TMDS-BMI: IR (KBr) 2,950 (aliphatic C-H), 1,700 (maleimide C=O), 1,260 (Si-C), 1,045 (Si-O), 790 (Si-C); ^1^H-NMR (CDCl_3_, 400 MHz) *δ* = 6.69 (s, 4H, maleimide), 3.41 (t, 4H, C*H*_2_-N<), 1.58 (m, 4H, -C*H*_2_-), 0.47 (t, 4H, C*H*_2_-Si≡), 0.04 (s, 12H, C*H*_3_-Si).

### 2.4. Diels-Alder polymerization of DFTreh and DMS-BMI

The Diels-Alder reaction was carried out in test tubes with a diameter of 30 mm in a personal organic synthesizer, ChemiStation PPS-5510 (EYELA, Japan), for controlling the temperature. To a solution of DFTreh (0.10 g, 1.9 mmol) in 3 mL of DMF or dioxane was added DMS-BMI (TMDS-BMI, DMS20-BMI, or DMS40-BMI; each 1.9 mmol). DMF was used for TMDS-BMI and DMS20-BMI; and dioxane for DMS40-BMI. The solution was stirred at prescribed temperature under a nitrogen atmosphere. After the reaction, the solution was added to 40 mL of water, and resulting precipitate was dried at room temperature for 24 h to yield a brown powder. The products from TMDS-BMI, DMS20-BMI and DMS40-BMI were denoted as DFT-TMDS-BMI, DFT-DMS20-BMI and DFT-DMS40-BMI, respectively. For DFT-TMDS-BMI: IR (KBr): 3,500 (OH), 2,950 (CH), 1,700 (imide C=O), 1,260 (Si-C), 1,060, 980 (Si-O, acetal, C-O), 800 (Si-C); ^1^H-NMR (DMF-*d_7_*, 400 MHz) *δ* = 6.6–6.3 (m, 4H, -C*H*=), 5.4–5.0 (m, 10H, >C*H*-O-, O*H-*3, acetal-*H*, H*-1*, O*H-2*), 4.2–3.9 (m, overlapping with resonance of water, 4H, *H-5, H-6-1*), 3.8–3.5 (m, 4H, *H-3*, *H-6-2*), 3.5–3.2 (m, 8H, *H-2*, *H-4*, >N-C*H*_2_-), 3.2–2.9 (m, 4H, >N-C(=O)-C*H*<), 1.6 (broad, 4H, -C*H*_2_-), 0.5 (broad, 4H, -C*H*_2_-Si), 0.06 (d, 12H, C*H*_3_-Si).

### 2.5. Retro-Diels-Alder reaction of the polymer product from DFT-DMS20-BMI

The retro-Diels-Alder reaction in solution state was carried out as follows. A solution of 100 mg of DFT-DMS20-BMI in 5 mL of DMF was heated to 100 °C using the ChemiStation PPS-5510, and the temperature was kept at 100 °C for 6 h. After the temperature reached 100 °C, 100 μL aliquots of the solution was taken out at 1 h intervals and diluted with 1 mL of DMF. The diluted sample was analyzed by gel permeation chromatography (GPC).

### 2.6. Characterization

Fourier transform infrared (FT-IR) spectra were recorded on a Shimadzu FT-IR 8100 by the KBr-pellet method and the attenuated total reflectance (ATR) method using a MIRacle attachment with a ZnSe prism. Proton nuclear magnetic resonance (^1^H-NMR) spectra were recorded on a Bruker AV-400 (400 MHz) using DMSO-*d*_6_, CDCl_3_ or DMF-*d*_7_ as a solvent. GPC of the polymer products was carried out at 40 °C on a Shodex GPC apparatus equipped with two SB-806M HQ GPC columns (Showa Denko K. K.) and a reflective index detector. DMF was used as the eluent at a flow rate of 0.5 mL/min. Polystyrene standards with a narrow distribution of molecular weight (*M*_w_: 580–377,400) were used for molecular weight calibration. The solubility of polymer products was tested by suspending 5 mg of the polymer powder in 0.5 mL of solvent. The mixture was sonicated for 1 min and allowed to stand for 24 h at room temperature.

The tensile test was carried out by a Shimadzu EZ-S tabletop universal tester with a 50 N load cell at a crosshead speed of 5 mm/min using rectangular specimens with width of 5 mm, length of 35 mm and thickness of ca. 25 μm. In each case, five specimens were tested and the average values of properties were taken.

Thermogravimetric analysis (TGA) was conducted with a Perkin Elmer TGA 7 thermogravimetric analyzer, at a heating rate of 20 °C/min under a nitrogen atmosphere. The thermal degradation temperature (*T*_d_) was defined as the temperature at which the weight of the sample decreased by 5%. Differential scanning calorimetry (DSC) was performed on a Perkin Elmer differential scanning calorimeter, Diamond DSC, under a helium atmosphere using a cooling attachment with liquid nitrogen. The sample for DSC was weighed (~5 mg) on a small aluminum pan, followed by sealing the pan. The sample was heated from -150 °C to 180 °C at a heating rate of 20 °C/min. The glass transition temperature (*T*_g_) was determined from the inflection point of the heat flow curve. Dynamic mechanical analysis of the rectangular sample (length 40 mm, width 7 mm, thickness 0.3 mm) cut from the polymer films was performed on a Rheolograph Solid dynamic mechanical analyzer (Toyoseiki Co., Ltd., Tokyo, Japan) with a stretching platform placed at an interval of 20 mm at a frequency of 10 Hz. The sample was heated from -150 °C to 100 °C at a heating rate of 2 °C/min.

The nano-order morphology of the polymer was observed by a Hitachi H-7100FA field emission transmission electron microscope (FE-TEM). Thin films were prepared by a cryo-microtome and set on a grid. The acceleration voltage was 100 kV.

## 3. Results and Discussion

### 3.1. Synthesis of DFTreh and DMS-BMI

**Figure 1:**
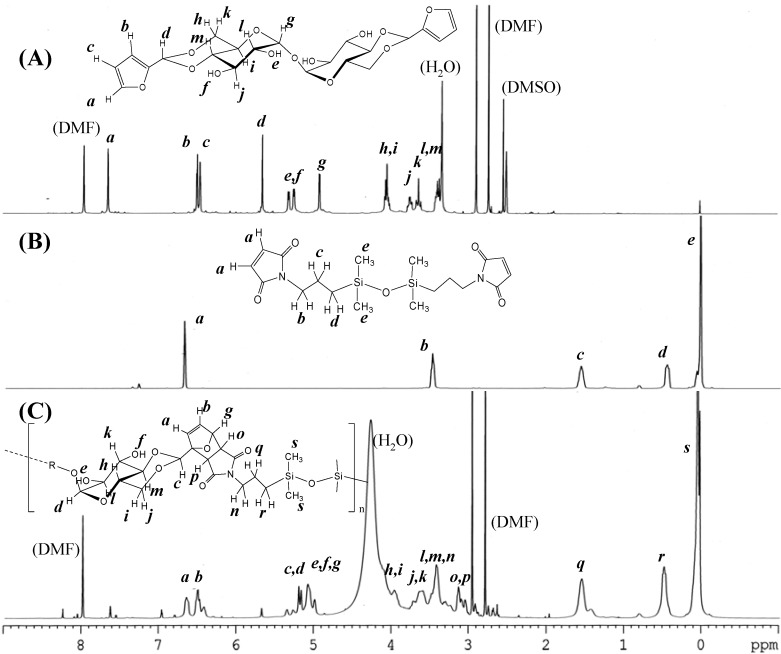
^1^H-NMR spectra of (A) 4,6,4',6'-*O*-difurfulylidene-α,α-d-trehalose (DFTreh), (B) bis(3-maleimidopropyl)tetramethyldisiloxane (TMDS-BMI), and (C) the polymer product, DFT-TMDS-BMI.

The synthesis of DFTreh involves the acetalization reaction of a d-glucopyranose unit with furfural in the presence of sulfonic acid-type cation exchange resin. In our previous report, the reaction occurs regioselectively on the 4,6-OH groups of the d-glucopyranose units of trehalose [6]. An excess amount of furfural was used for completion of the acetalization reaction. The ^1^H-NMR spectrum of the product is shown in Figure 1 (A). All signals correspond to those in our previous report. The methine signal adjacent to the furan ring appeared at 5.65 ppm (d), indicating successful production of the acetal.

The synthesis of DMS-BMI involves the condensation reaction of an amine and maleic anhydride. This is a two-step reaction with an intermediate compound having maleamic acid groups. The ^1^H- NMR spectrum of the product of maleic anhydride and TMDS-BA is shown in Figure 1 (B). The signal at 6.69 ppm (a) was assigned to the maleimide group methine protons; the signals at 3.41, 1.58 and 0.47 ppm (b, c, d) to the propyl group methylene protons. The large signal at 0.04 was assigned to the methyl protons of the dimethylsiloxane units. These results suggest that the desired products were obtained.

### 3.2. Synthesis of trehalose-based polymers by the Diels-Alder reaction

The Diels-Alder reaction of DFTreh and bismaleimides was reported previously [6]. The reaction proceeds by gentle heating under 90 °C; and if the temperature over 90 °C, the reverse reaction, *i.e.,* the retro-Diels-Alder reaction competes against the forward reaction. In the present study, the reaction temperature was varied from 40 °C to 70 °C. When we examined the reaction time of 24 h and 48 h at 55 °C, the molecular weight of the product obtained from longer reaction time was higher. The weight average molecular weight (*M*_w_) of DFT-TMDS-BMI was 8,500 in the 24-h reaction at 55 °C, while *M*_w_ was 11,000 in the 48-h reaction. The reaction time was fixed as 48 h below. Experimental data for polymer syntheses were summarized in Table 1.

**Table 1:** Diels-Alder polymerization of DFTreh with siloxane oligomers. DMF was used for TMDS-BMI and DMS20-BMI; and dioxane for DMS40-BMI.

Product	Reaction condition		Yield (%)	Molecular weight (GPC)			
	Temperature (°C)	Time (h)		*M*_w_	*M*_n_	*M*_w_/*M*_n_	DP ^*^
DFT-TMDS-BMI	40	48	49	7,100	2,200	3.2	2.4
DFT-TMDS-BMI	55	48	47	11,000	2,900	3.8	3.2
DFT-TMDS-BMI	70	48	51	14,000	3,300	4.2	3.6
DFT-DMS20-BMI	40	48	69	8,900	4,000	2.2	1.8
DFT-DMS20-BMI	55	48	63	18,000	7,500	2.4	3.4
DFT-DMS20-BMI	70	48	60	19,000	6,800	2.8	3.0
DFT-DMS40-BMI	40	48	48	9,600	3,200	3.0	0.9^†^
DFT-DMS40-BMI	55	48	82	13,000	9,000	1.4	2.4
DFT-DMS40-BMI	70	48	62	7,000	3,500	2.0	0.9^†^

* Degree of polymerization (DP) was calculated from *M*_n_; ^†^ The monomer peak was overlapped with the polymer peak in the GPC chart.

In the synthesis of DFT-TMDS-BMI, the molecular weight increased with an increase of the reaction temperature; and in the syntheses of DFT-DMS20-BMI and DFT-DMS40-BMI, the molecular weight was saturated or maximized at 55 °C. The polydispersion index (*M*_w_/*M*_n_) decreased at the increasing length of oligo(dimethylsiloxane) units, because degree of polymerization (DP) was low. The low DP observed in the polymer with large oligo(dimethylsiloxane) units (DFT-DMS40-BMI) is attributed to lowered concentration of reactive functional groups. However, *M*_w_ of most polymers obtained in the reaction at 55 °C and 70 °C for 48 h exceeds 10,000.

Figure 1 (C) shows the ^1^H-NMR spectra of reactants and the product, DFT-TMDS-BMI. The methine signals adjacent to the carbonyl groups of imide appeared at 3.2–2.9 ppm (o, p); and the methine signals of dihydrofuran groups at 6.6–6.3 ppm (a, b) and 5.1 ppm (g). On the other hand, the methine signals of maleimide at 6.9 ppm and those of furan groups at 7.6 and 6.4 ppm diminished in the products, though they did not disappear completely.

### 3.3. Solubility of polymers

**Figure 2:**
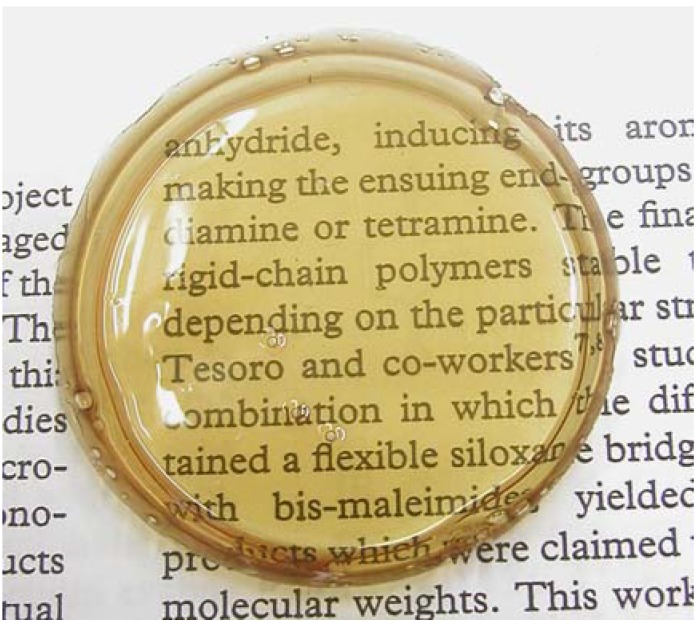
Photograph of the as-cast film of DFT-DMS20-BMI.

Table 2 shows the results of the solubility tests of each polymer in various solvents. Polymers were soluble in many organic solvents; especially in polar solvents such as chloroform, tetrahydrofuran, and DMF. Solubility in dimethylsulfoxide (DMSO), which is a highly polar solvent, reflects the relative hydrophilicity of the polymers: DFT-TMDS-BMI was soluble in DMSO, DFT-DMS20-BMI was partially soluble, and DFT-DMS40-BMI was insoluble. On the other hand, solubility in ethyl acetate, toluene and diethylether reflects the hydrophobicity: DFT-TMDS-BMI was insoluble in any of these three solvents; DFT-DMS20-BMI was insoluble in diethylether but soluble in ethyl acetate and toluene; and DFT-DMS40-BMI was soluble in any of these three solvents. Flexible brown transparent films (Figure 2) can be obtained by casting from the chloroform solutions of DFT-DMS20-BMI and DFT-DMS40-BMI. DFT-TMDS-BMI was so brittle that it could not form a film.

**Table 2:** Solubility of the polymers (i, insoluble; ps, partially soluble; s, soluble).^*^

Solvent	DFT-TMDS-BMI	DFT-DMS20-BMI	DFT-DMS40-BMI
Water	i	i	i
Methanol	i	i	i
Ethanol	i	i	i
Dimethylsulfoxide	s	ps	i
*N*,*N*-dimethylacetamide	s	s	ps
DMF	s	s	s
Acetone	ps	s	s
Dioxane	s	s	s
Tetrahydrofuran	s	s	s
Chloroform	s	s	s
Ethyl acetate	i	s	s
Toluene	i	s	s
Diethylether	i	i	s
Hexane	i	i	i

* Solubility test was carried out by suspending polymer powder in a solvent by sonication for 1 min and allowing it to stand for 24 h at room temperature.

### 3.4. Thermal properties of polymers

**Figure 3:**
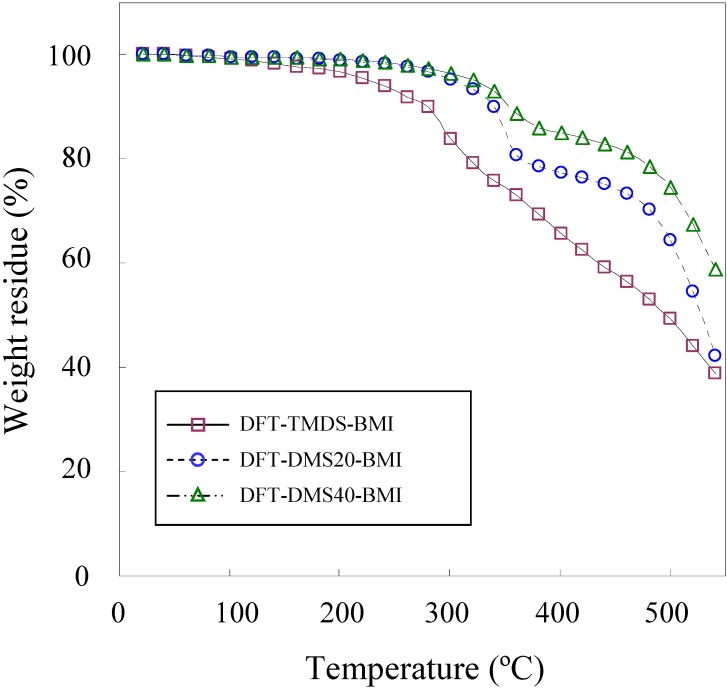
TGA curves of DFT-TMDS-BMI, DFT-DMS20-BMI and DFT-DMS40-BMI, measured under a nitrogen atmosphere.

The thermal degradation temperature (*T*_d_) was measured by TGA and the glass transition temperature (*T*_g_) was measured by DSC. Figure 3 shows the TGA weight loss curves of polymers. Among three polymers synthesized in the present study, DFT-TMDS-BMI has the lowest *T*_d_. The *T*_d_ of the polymer was greatly influenced by the size of the oligo(dimethylsiloxane) unit. DFT-DMS20-BMI and DFT-DMS40-BMI exhibited relatively high *T*_d_, because these polymers containing large siloxane blocks. The *T*_d_ (5% weight loss) of monomers DFTreh and TMDS-BMI were 245 °C and 232 °C, respectively, while the *T*_d_ of DMS20-BMI and DMS40-BMI were 310 °C and 416 °C, respectively. This is due to the higher chemical stability of Si-O bonds than C-C bonds and C-O bonds [26]. TGA curves of DFT-DMS20-BMI and DFT-DMS40-BMI exhibited definitely two-step decomposition profiles, and very similar profiles were observed in the TGA measurement of DMS20-BMI and DMS40-BMI. The first decomposition step occurred at around 350 °C, which may correspond to the decomposition of organic segments of polymers; and the second step at around 500 °C, which may correspond to that of dimethylsiloxane segments. The weight residues at 420 °C were 62.5%, 76.3%, 84.1%, and 29.7% for DFT-TMDS-BMI, DFT-DMS20-BMI, DFT-DMS40-BMI, and DFTreh, respectively. The result means that the percent degradation values were 37.5%, 23.7%, 15.9%, and 70.3%, respectively. Considering that the organic segments were degraded at the first decomposition step, the organic contents were calculated to be 53.3% for DFT-TMDS-BMI (theoretically 82.3%), 33.7% for DFT-DMS20-BMI (theoretically 33.3%), and 22.6% for DFT-DMS40-BMI (theoretically 20.0%). The TGA measurement is based on the weight loss by the vaporization of thermally degraded products with low molecular weight. The difference from the theoretical value, especially seen for DFT-TMDS-BMI, may be due to either the retardation of the degradation of the polymer chain or the interference of the vaporization of the degraded products, though the reason is unclear.

**Figure 4:**
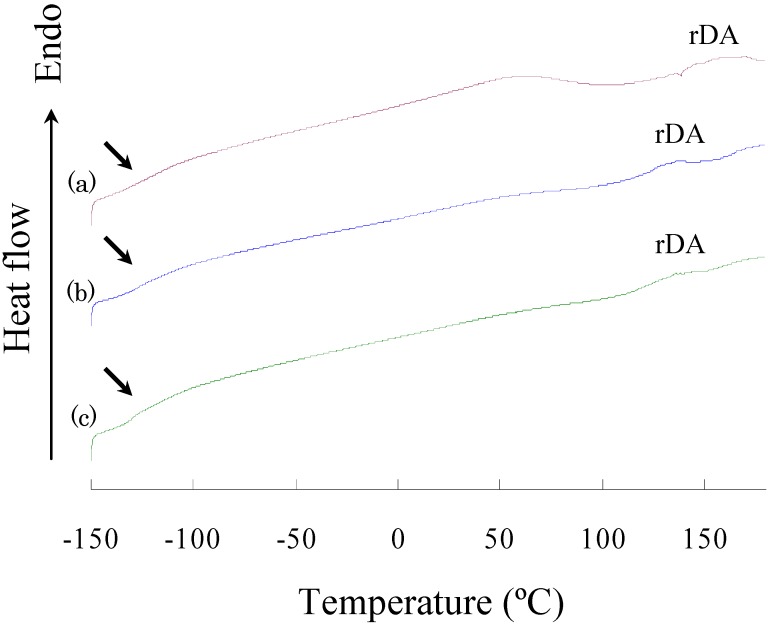
DSC curves of (a) DFT-TMDS-BMI, (b) DFT-DMS20-BMI, and (c) DFT-DMS40-BMI. Arrows indicate the lower glass transition temperatures; and "rDA" indicates the retro-Diels-Alder reaction.

Figure 4 shows the DSC heat flow curves of polymers in the heating process. Three polymers exhibited *T*_g_ at -119~-130 °C and a broad endothermic peak at 110~160 °C. The former corresponds to *T*_g_ of oligo(dimethylsiloxane) segments, and the latter may correspond to the retro-Diels-Alder reaction [6,25]. The heat capacity gap (*ΔC*_p_) at the *T*_g_ increased with the size of oligo(dimethylsiloxane) units, and the endothermic enthalpy (*ΔH*) at the retro-Diels-Alder reaction decreased with the size of oligo(dimethylsiloxane) units (Table 3). Since the oligo(dimethylsiloxane) segments are known to be amorphous and the polymers exhibited very low *T*_g_, the polymers would be liquid at room temperature if they were consist of uniform phase. Considering that these polymers are solid at room temperature, the phase-separated structure is expected to be formed: the first phase is the rubber phase consisting of a soft segment region of oligo(dimethylsiloxane) units as a matrix, and the second phase is the glass phase consisting of a hard segment region of trehalose and tricyclodecene structures. The hard segments impart the partial rigidity to the flexible polymer chain and play a role as a crosslinking section with hydrogen bonds. In the previous study, we observed two glass transitions corresponding to the two phases in the DSC measurement of trehalose-based linear polymers containing oligo(dimethylsiloxane) units [7]: the lower *T*_g_ was around -110 °C, and the higher varied from 96 °C to 152 °C depending on the length of the oligo(dimethylsiloxane) units. However, we could not observe a glass transition at a temperature higher than the room temperature in the DSC measurement in the present study. The DSC curves showed a broad endothermic peak at 110~160 °C corresponding to the retro-Diels-Alder reaction, and this peak most likely overlapped with the higher *T*_g_ corresponding to the transition of the hard segments. Moreover, because the retro-Diels-Alder reaction causes the scission of the polymer main chain, it really hid the higher *T*_g_. A broad endothermic peak at 50~70 °C observed in the DSC curve of DFT-TMDS-BMI is unexplained. The peak is considered to originate from the vaporization of a trace of water which was adsorbed onto the polymer surface during handling.

**Table 3:** Thermal property data drawn from the DSC measurements.

Product	*T_g_* ( °C)	*ΔC*_p_ at *T_g_* (J/g•K)	Peak temperature at rDA (°C)	*ΔH* at rDA (J/g)
DFT-TMDS-BMI	-119	0.194	158	14.3
DFT-DMS20-BMI	-128	0.306	136	7.48
DFT-DMS40-BMI	-127	0.367	136	5.62

**Figure 5:**
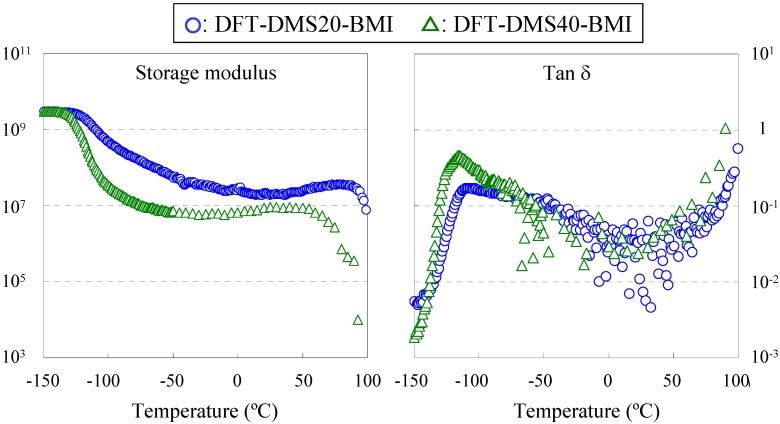
Dynamic mechanical analysis of DFT-DMS20-BMI and DFT-DMS40-BMI.

Dynamic mechanical measurement is the method for determining relaxations, and the alpha relaxation is related to *T*_g_. We tried to determine *T*_g_ by dynamic mechanical measurement of DFT-DMS20-BMI and DFT-DMS40-BMI. The measurement of DFT-TMDS-BMI was impossible because a stand-alone film was not obtained, and the measurement of DFT-DMS20-BMI and DFT-DMS40-BMI over 100 °C was also impossible because of the sample weakening. Figure 5 shows the storage modulus (E') and the tanδ curves vs. temperature. The storage modulus was greatly influenced by temperature. The first depression of the storage modulus of DFT-DMS40-BMI occurred at the lower *T*_g_ at -115 °C (tan δ peak); and the second depression started at 60 °C. Similarly, the first depression of the storage modulus of DFT-DMS20-BMI occurred at the lower *T*_g_ at -108 °C (tan δ peak); and the second depression started at 90 °C. The DSC measurement revealed, as described previously, that the retro-Diels-Alder reaction start at 110 °C, and the sample rapidly softened over 100 °C. Therefore we could not determine the (hypothetical) higher *T*_g_. We postulated that the second depression of the storage modulus was related to the higher *T*_g_, and found that the temperature depends on the length of the oligo(dimethylsiloxane) units. If the each phase were separated completely, the *T*_g_ would not depend on the length of the oligo(dimethylsiloxane) units. To explain this shift of the dependency, we should picture the partial phase separation [7], especially in the region of trehalose with tricyclodecene units. The discussion on the phase separation structure is continued in the next section.

### 3.5. Morphology of polymers

**Figure 6:**
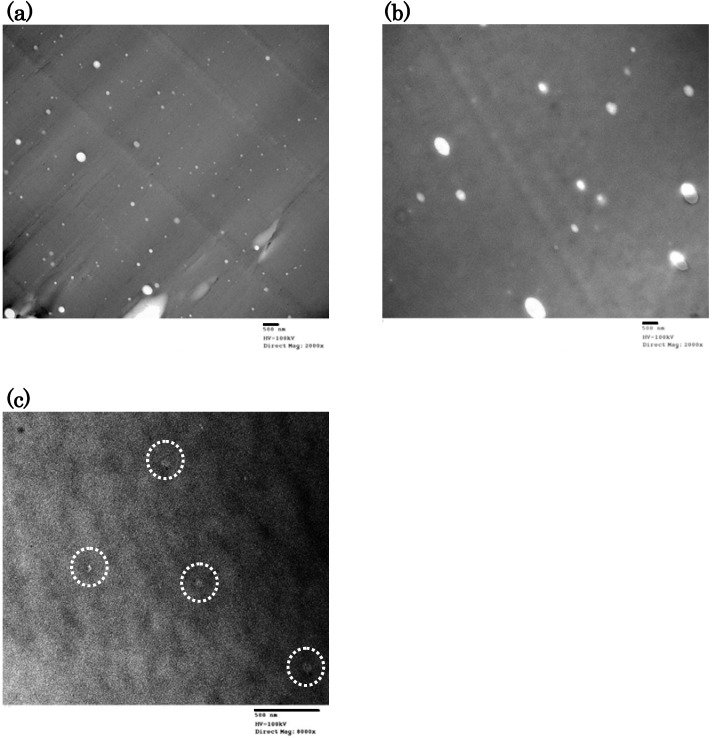
TEM photographs of (a) DFT-TMDS-BMI, (b) DFT-DMS20-BMI, and (c) DFT-DMS40-BMI. The acceleration voltage was 100 kV. The obscure small spots are highlighted in the photograph (c). The scale bar under each photograph represents 500 nm.

At first, we tried to observe the morphology of polymers by FE-SEM. However, the FE-SEM photographs showed very smooth polymer surfaces and the phase-separation morphology was not observed. Next, we carried out the TEM observation of the cryo-microtome section of the polymer. Figure 6 shows the TEM photographs of DFT-TMDS-BMI, DFT-DMS20-BMI and DFT-DMS40-BMI. Many white spots dispersed in the dark matrix were observed in DFT-TMDS-BMI and DFT-DMS20-BMI, though only a few obscure smaller spots located in the matrix were observed in DFT-DMS40-BMI. The dispersed phase of white spots corresponds to trehalose units and the dark matrix phase corresponds to siloxane units, because Si atoms have higher electron density. The morphology of DFT-DMS40-BMI was obscure due to the low content of trehalose units. The partial phase separation of trehalose units described above also promoted the obscuration. The size of white spots observed in DFT-DMS20-BMI is larger than in DFT-TMDS-BMI, although white spots were observed more abundantly in DFT-TMDS-BMI.

### 3.6. Mechanical properties of polymers

**Figure 7:**
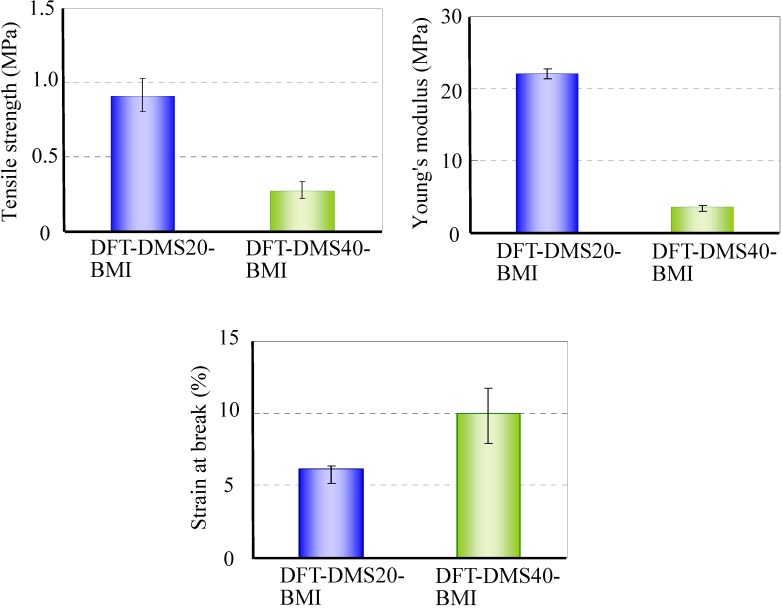
Tensile properties of DFT-DMS20-BMI and DFT-DMS40-BMI.

A tensile test of DFT-DMS20-BMI and DFT-DMS40-BMI was carried out. We could not carry out the tensile test on DFT-TMDS-BMI because of its brittleness. Figure 7 shows the tensile strength, Young's modulus, and the strain at break. The tensile strength and Young's modulus of DFT-DMS20-BMI were higher than those of DFT-DMS40-BMI, and the strain at break of DFT-DMS40-BMI was higher than that of DFT-DMS20-BMI. The results suggest that oligo(dimethylsiloxane) units afford the flexibility to the polymer. On the other hand, because the hard segments of trehalose and tricyclodecene play a role as a crosslinking section, the decrease of the hard segments leads to weakening of the polymer strength.

### 3.7. Retro-Diels-Alder reaction

**Figure 8:**
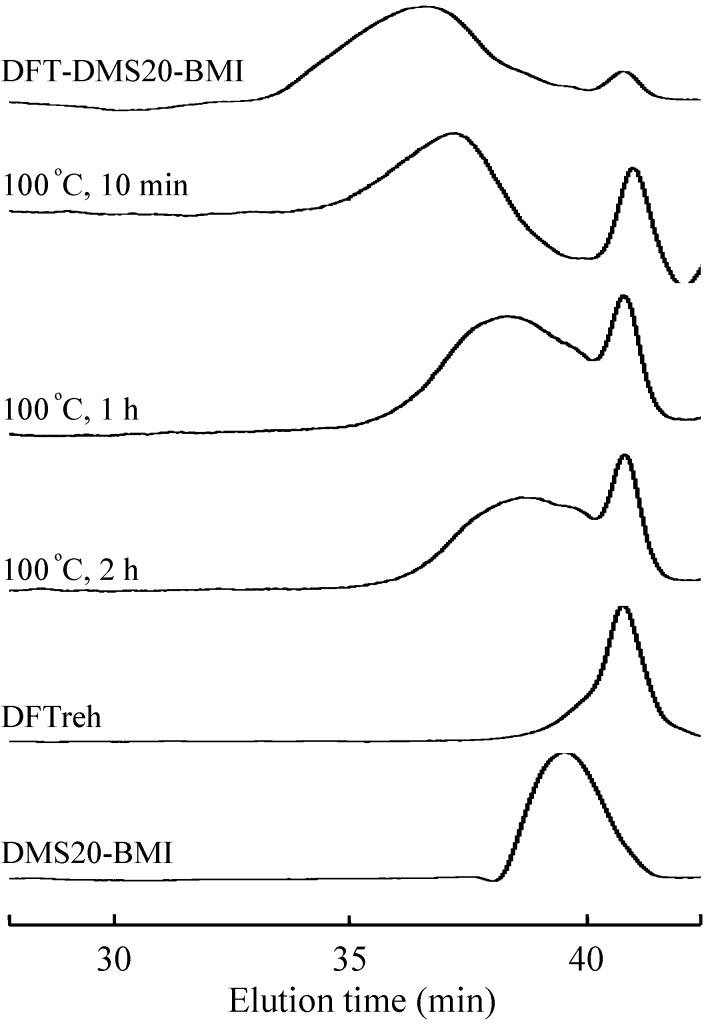
GPC traces of DFT-DMS20-BMI kept heated at 100 °C in DMF for the indicated time.

The retro-Diels-Alder reaction is the reverse reaction of the Diels-Alder reaction, in which the tricyclodecene adduct degrades into diene and dienophile. In our previous report and the preliminary test, the adduct of furan with maleimide was found to degrade at ~100 °C in solution and at ~130 °C in solid form [6]. DFT-DMS20-BMI was dissolved in DMF, and the solution was kept at 100 °C. The aliquot of the solution was analyzed by GPC at the prescribed time and the GPC charts were shown in Figure 8. Obviously, the elution time of the polymer was delayed with an increase of reaction time. This result indicates that the molecular weight of the polymer decreased by heating at 100 °C. Furthermore, the elution peak at 40 min increased in the course of the reaction. The emerging peak corresponds to the peak of DFTreh, implying that the retro-Diels-Alder reaction proceeded at 100 °C to yield monomers. However, the broad elution peak at 39 min contains longer molecules than DMS20-BMI, and the peak did not shift in the further reaction at 100 °C. This result suggests that some side reactions occurred at 100 °C, especially involving the BMI monomer.

## 4. Conclusions

4,6,4',6'-*O*-Difurfurylidene-α,α-d-trehalose (DFTreh) was synthesized by the acetalization of trehalose with furfural, both of which are renewable resources. Maleimide-terminated dimethylsiloxane oligomers (DMS-BMI) with different length were synthesized from corresponding amine-terminated dimethylsiloxane oligomers and maleic anhydride. The Diels-Alder reaction of DFTreh with DMS-BMI in DMF afforded alternating copolymers composed of trehalose units and oligo(dimethylsiloxane) units in 47–82% yield. The *M*_w_ measured by GPC of the polymers ranged from 7,000 to 19,000. While DFT-TMDS-BMI was soluble in DMSO and insoluble in toluene, DFT-DMS40-BMI was insoluble in DMSO and soluble in toluene. The size of the oligo(dimethylsiloxane) unit influenced hydrophobicity of the polymer. Flexible transparent films were obtained by casting from the chloroform solutions of DFT-DMS20-BMI and DFT-DMS40-BMI. *T*_d_ was also greatly influenced by the size of the oligo(dimethylsiloxane) unit. The polymer having longer oligo(dimethylsiloxane) units shows the higher *T*_d_. Each polymer shows a glass transition at -119~-130 °C and a broad endothermic peak at 110~150 °C in the DSC measurement. The broad endothermic peak corresponds to the retro-Diels-Alder reaction. The *T*_g_ was also observed in the dynamic mechanical analysis as the tan δ peak. The polymer microtome sections were observed by TEM, and the polymers were found to have a phase-separated morphology. GPC analysis following the thermal incubation of the polymer solution at 100 °C revealed that the monomer was recovered by the retro-Diels-Alder reaction. However, the possibility of side reactions during retro-Diels-Alder reaction cannot be excluded.
